# Description of the male of *Craugastor yucatanensis* (Lynch, 1965) (Anura, Craugastoridae), its advertisement call, and additional data on females

**DOI:** 10.3897/zookeys.900.37591

**Published:** 2019-12-31

**Authors:** Rubén Alonso Carbajal-Márquez, Luis F. Díaz-Gamboa, Tania Ramírez-Valverde, Christian M. García-Balderas, Pedro E. Nahuat-Cervera, José Rogelio Cedeño-Vázquez

**Affiliations:** 1 El Colegio de la Frontera Sur, Departamento de Sistemática y Ecología Acuática, Unidad Chetumal, Avenida Centenario Km 5.5, 77014, Chetumal, Quintana Roo, México El Colegio de la Frontera Sur (ECOSUR) Chetumal Mexico; 2 Conservación de la Biodiversidad del Centro de México, A.C. Andador Torre de Marfil No. 100, 20229, Aguascalientes, Aguascalientes, México Conservación de la Biodiversidad del Centro de México Aguascalientes Mexico; 3 Universidad Autónoma de Yucatán, Campus de Ciencias Biológicas y Agropecuarias, km. 15.5 Carr. Mérida-Xmatkuil, 97315, Mérida, Yucatán, México Red para la Conservación de los Anfibios y Reptiles de Yucatán (RCARY) Sierra Papacal Mexico; 4 Red para la Conservación de los Anfibios y Reptiles de Yucatán (RCARY), Carretera Sierra Papacal-Chuburna Puerto Km. 5, Sierra Papacal, Yucatán, México Universidad Autónoma de Yucatán Mérida Mexico

**Keywords:** Natural history, sexual dimorphism, vocalization, Yucatán Rainfrog

## Abstract

The male of *Craugastor yucatanensis* (Lynch, 1965) is described for the first time, as the original description was based on four females. The advertisement call is described and additional morphological data on females are presented. Also, information is provided on the sexual dimorphism and natural history of the species.

## Introduction

Lynch (1965) described *Craugastor yucatanensis* from a cave near Nuevo Xcán, Quintana Roo, Mexico, attributing the specific name to the Yucatán Peninsula. However, the description was based exclusively on four females. *Craugastor yucatanensis* is a member of the Craugastor (Hylactophryne) bocourti species series (sensu Hedges et al. 2008), which includes 19 species. Subsequently, Padial et al. (2014) rejected species groups within the subgenus Hylactophryne. *Craugastor yucatanensis* inhabits the central and northeastern parts of the Yucatán Peninsula and is the only member of the genus found in the Mexican portion of this peninsula (Lee 1996; Köhler 2011; González-Sánchez et al. 2017). In recent years we have observed and collected specimens of both sexes of *C. yucatanensis* near the type locality, and reviewed museum specimens from additional localities. We describe herein the previously unknown male and advertisement call, and present morphological data on females. Also, we provide information on sexual dimorphism and natural history.

## Materials and methods

We conducted field trips in 2015–2018 to locate individuals of *C. yucatanensis* in the vicinity of the type locality at Nuevo Xcán, Quintana Roo, Mexico. Specimens used in the description are deposited in the herpetological collection of El Colegio de la Frontera Sur (ECOSUR) at Chetumal, Quintana Roo, Mexico (ECO-CH-H). We followed Lynch’s (1965) measurements from the species description. Abbreviations used are: SVL (snout-vent length), TM (tympanum length), EL (eye length), IOD (interorbital distance), SL (tibia segment), F3 (width of pad of 3^rd^ finger), EN (eye to nostril distance), HW (head width), and HL (head length). The measurements follow Duellman (1970), expressed in millimeters, to the nearest 0.1 mm, and were obtained by a digital caliper (Mitutoyo). Adult specimens included in the measurement series were maintained in 70% ethyl alcohol. Male specimens were identified at the time of collection based on advertisement call. The sex of preserved specimens was determined by the presence of white prepollical nuptial excrescences in adult males and their absence in females. Color descriptions of live specimens were based on Köhler (2012). Color descriptions in the diagnoses refer to live specimens. We calculated the mean, standard deviation, and range for each morphometric variable. We tested differences between males and females in SVL with the Student *t*-test, after testing variables for normality with the Kolmogorov-Smirnov test. We tested differences in TM, EL, ID, SL, F3, EN, HL and HW with analyses of covariance, with SVL as the covariate, and sex as a dependent variable. All variables were log transformed, and all statistical analyses were performed in Statistica (StatSoft Inc., Tulsa, Oklahoma, USA), with a statistical significance threshold of *P* ≤ 0.05.

We recorded advertisement calls of male frogs while they were actively calling in the field, using the WavePad free recording software (NCH Software 2015) on a Samsung Galaxy J7 smartphone with an internal directional microphone. We recorded the calls at distances of 50–150 cm. Digital sonograms were executed to identify the frequencies emitted, as well as determine the other sound sources that also formed part of the landscape. We selected the frequencies of the species to later filter unwanted frequencies through multiple parametric equalizers using the Ableton Live 10 program (Ableton 2017). Finally, the recordings were edited to emphasize the time cycles of the species and create a one-minute sample with the following sampling rate: 48,000 hertz, 2,880,000 samples, and 24 bits of resolution. We obtained a frequency spectrogram using the “seewave” version 1.6.4 package (Sueur et al. 2008) of R version 3.5.0, 64-bit version (R Core Team 2018). The “seewave” settings were as follows: window name (Fast Fourier Transform window) = Hanning; window length = 512 samples; sampling rate = 48,000 hertz; number of samples = 2,880,000; and overlap = 80%.

## Results

### Craugastor yucatanensis

Taxon classificationAnimaliaAnuraCraugastoridae

(Lynch, 1965)

41977646-D6A4-57D3-924D-320D35653118

#### Material examined.

***Craugastor yucatanensis*** (23). Mexico – **Quintana Roo State**: Benito Juárez Mun. ECO-CH-H-1655; Felipe Carrillo Puerto Mun. ECO-CH-H-1878, 1904, 1932, 1949, 2014, 2015, 2016, 2042, 2105, 2393, 3538 –**Yucatán State**: Chemax Mun. ECO-CH-H-3790, 3791, 3792, 3793, 4537, 4538, 4539, 4540, 4541, 4542; Tinum Mun. ECO-CH-H-3539.

#### Diagnosis.

**Figure 1.**
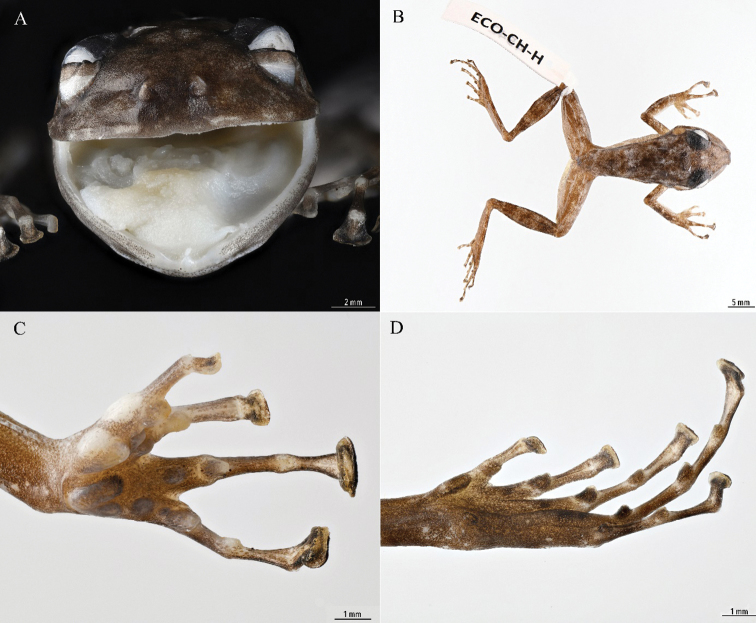
Male of *Craugastor yucatanensis* (ECO-CH-H-4542) view inside of mouth (**A**) in dorsal aspect (**B**) ventral view of hand (**C**) and ventral view of foot (**D**). Photos by Humberto Bahena Basave.

A member of the Craugastor (Hylactophryne) bocourti species series, most closely related to *C. alfredi* (Boulenger, 1898) (Lynch, 1965), characterized by having greatly expanded and truncate digital pads on the outer two fingers, possessing no vocal sac or slits (Fig. 1A), a relative large tympanum in adult males (74.7% [65.7–85.5%] of eye diameter) and females (52.4% [42–65.5%] of eye diameter), canthus rostralis rounded; supernumerary tubercles on palm and sole; venter semi-transparent or pinkish; finger pads large, slightly emarginate, and having a fine tarsal ridge. Differs from the closest related species within the series lacking vocal sacs (e.g., *C. alfredi*, *C. campbelli* (Smith, 2005), *C. cyanochthebius* McCranie & Smith, 2006, *C. galacticorhinus* (Canseco-Márquez & Smith, 2004), *C. glaucus* (Lynch, 1967), *C. megalotympanum* (Shannon & Werler, 1955), *C. nefrens* (Smith, 2005), *C. stuarti* (Lynch, 1967), *C. taylori* (Lynch, 1966), and *C. xucanebi* (Stuart, 1941)) by a relatively larger tympanum in males – 74.7% of eye diameter (versus about two-thirds of eye diameter), supratympanic fold absent (versus poorly developed), and numerous small tubercles on the sole (versus few or absent).

#### Description and variation of males.

Adult males (*N* = 19) averaged SVL = 27.1 ± 1.7 mm (range 24.2–30.5 mm). Head somewhat broader (HW = 11.2 ± 0.9 mm [9.6 – 13.2]) than long (HL = 10.5 ± 0.6 mm [9.7–12.1]). Tympanum distinct, more than two-thirds the diameter of the eye (TM = 2.8 ± 0.2 mm [2.4–3.2]). Eye slightly longer (EL = 3.7 ± 0.3 mm [3.1–4.4]) than distance from EN = 3.6 ± 0.3 mm [3.1 – 4.0]). Average IOD = 3.4 ± 0.3 mm (2.8–4.0). Canthus rostralis rounded; loreal region slightly concave; tympanum transparent with a dark spot in the center; no dorsolateral, paravertebral, or occipital folds; supra and post-tympanic folds not distinct. Dorsum smooth; skin on venter smooth except for ventral surface of thigh which is areolate; scattered melanophores on chin, chest, and limbs; ventral disc present although obscure; posterior border of thighs slightly granular (Fig. 1B).

Tips of fingers expanded, slightly emarginate; the width of pad of 3^rd^ finger (F3) averages 1.6 ± 0.2 mm (1.2–2.1); thumb with an enlarged thenar tubercle, almost as large as semi-divided cordiform palmar tubercle; subarticular tubercles round to slightly obtuse and projecting in lateral profile, rounded to ovoid in basal outline, Fingers I and II with one, Fingers III and IV with two; accessory palmar tubercles globular to slightly conical in lateral profile, rounded in basal outline; first finger as long as second; with two white glandular nuptial pads, one on thenar tubercle and the other, also on pollex, opposite and slightly lateral to distal subarticular tubercle (Fig. 1C). Toes expanded slightly. Supernumerary tubercles on metatarsus; tarsal fold present for one-third length of tarsus; inner metatarsal tubercle elongate, not compressed; outer metatarsal tubercle present, round, diameter one-fifth length of inner metatarsal tubercle; subarticular tubercles globular (Fig. 1D). Tibia length averages 14.9 ± 0.7 mm (14.1–16.3). Heel reaching anterior edge of eye; legs held at a little less than a right angle to the body.

#### Description and variation of females.

Adult females (*N* = 8; including four from the original description) had an average SVL = 35.0 ± 1.9 mm (range 31.2–37.1 mm). Head is broader (HW = 14.1 ± 1.1 mm [12.5–15.1]) than long (HL = 13.1 ± 1.7 mm [11.8–17.1]). Tympanum distinct, half the diameter of the eye (TM = 2.5 ± 0.4 mm [2.0–3.1]). Eye slightly shorter (EL = 4.7 ± 0.4 mm [4.1–5.1]) than distance from EN = 4.9 ± 0.3 mm [4.4–5.3]). Average IOD = 4.0 ± 0.3 mm (3.4–4.5). Canthus rostralis rounded; loreal region slightly concave; no dorsolateral, paravertebral, or occipital folds; supra and post-tympanic folds not distinct. Tips of fingers expanded, slightly emarginate; the width F3 = 2.3 ± 0.4 mm (1.6–2.7). Tibia length averages 18.2 ± 1.0 mm (17.0–20.4).

#### Color in life.

**Figure 2.**
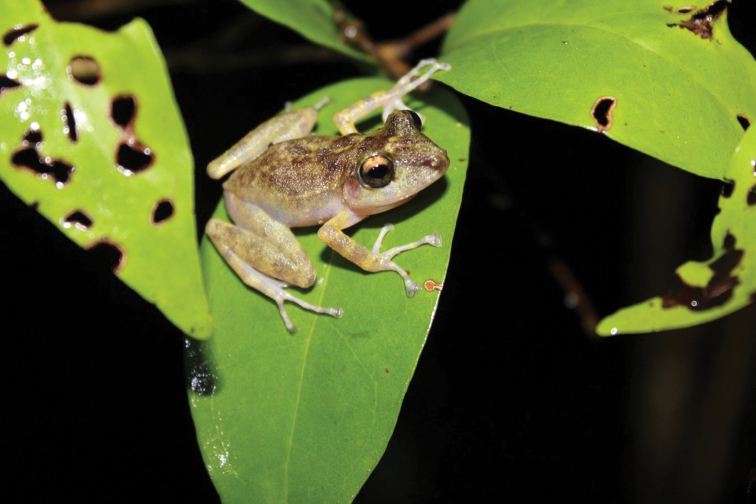
An adult male *Craugastor yucatanensis* found sitting on vegetation at Xcán, Yucatán, Mexico. Photo by Pedro E. Nahuat Cervera.

**Figure 3.**
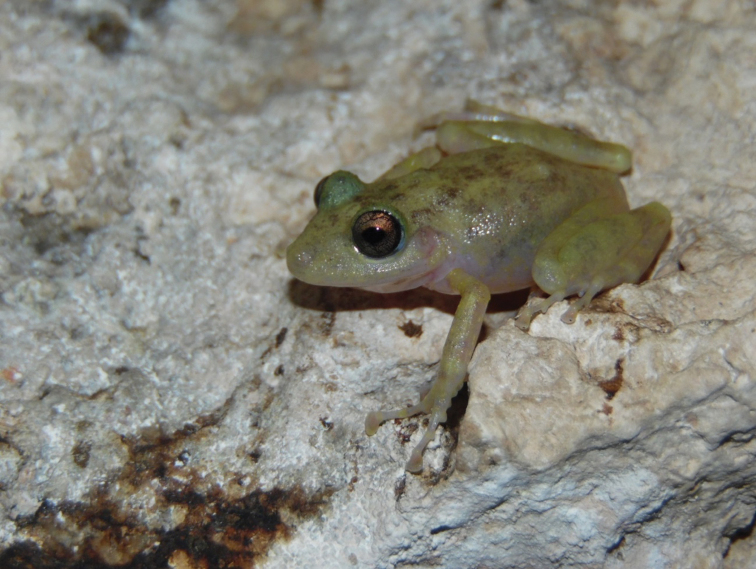
An adult female *Craugastor yucatanensis* found inside a cave at Opichén, Yucatán, Mexico. Photo by Pedro E. Nahuat Cervera.

The coloration depends on the substrate and the time when the specimens are found. When males are active at night over vegetation or leaf litter, they have an Olive Yellow (117) to Smoke Gray (267) dorsal coloration, with Glaucous (289) to Sepia (279) blotches on dorsum and bars on limbs; sometimes a thin clear vertebral stripe is distinguishable (Fig. 2). When found during the day in caves, they exhibit a coloration similar to that observed at night, but with a paler tone. When found during the day on leaf litter they have a Cinnamon-Rufous (31) dorsal coloration, where the blotches and bars are less evident. The dorsal coloration of females is Olive Yellow (117) to Smoke Gray (267) with scattered Glaucous (289) to Sepia (279) blotches when active at night, whether in caves or leaf litter. When found during the day inside caves, their coloration is paler (Fig. 3), sometimes becoming completely Pale Pinkish Buff (3), similar to some karstic limestone inside the caves. When they are found in leaf litter, their coloration is Cinnamon-Rufous (31), and in both cases the blotches and bars are little evident. The arms and legs are banded, the iris Pearl Gray (262) with metallic bronze tones, and the lateral and ventral surfaces are semi-transparent or Pinkish White (216) in both sexes.

#### Color in preservative.

Dorsum Pale Neutral Gray (296), with Glaucus (289) to Brownish Olive (292) blotches on dorsum and bars on limbs; pupil Smoky White (261), iris and upper eyelid Grayish Olive (273); tympanum Pale Cinnamon (55); venter semi-transparent or Pale Buff (1) to Pale Pinkish Buff (3) (Fig. 1B).

#### Advertisement call.

**Figure 4.**
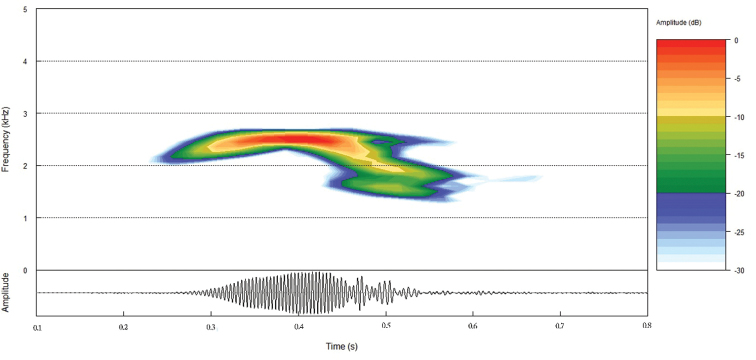
Spectrogram (top), and oscillogram (bottom) from a single note of the *Craugastor yucatanensis* advertisement call, recorded 11 km south of Nuevo Xcán, Yucatán, Mexico.

The advertisement call of *Craugastor yucatanensis* is part of a communication system that consists of repetitive notes emitted every 10 seconds (6 times per minute). Every note has a duration of approximately 460 MS at a dominant frequency around 2600 kHz. These notes sound like a very short “peep” that resembles the weak chirping of a bird chick (Fig. 4). The digital audio file can be accessed online at Díaz-Gamboa et al. (2019) at Soundcloud.

#### Distribution and natural history.

*Craugastor yucatanensis* is known from near sea level to 60 m elevation throughout its range on the central and northeastern portion of the Yucatán Peninsula (Lee 1996; Ortiz-Medina et al. 2016). The vegetation in this area is classified as tropical deciduous forest, low and medium semideciduous forest; high, medium and low semi-evergreen forest; tall evergreen forest, with karstic limestone outcroppings (Torrescano-Valle and Folan 2015). The males were located calling on 18–19 October 2016 and 22 July 2018, on vegetation 1.5–6.0 m above ground. Calling occurred at night (20:00–03:00 h) following afternoon or early evening rainfall, and during light rain later in the night. Many males could be heard calling from the vegetation. When we attempted to capture vocalizing males, they jumped to the ground where they jumped erratically, then stopped suddenly and became immobile in the leaf litter; crypsis was enhanced by rapid color change to darker tones (metachrosis). Additional males were found during the day and appeared to have been dislodged from a resting place in lower vegetation or leaf litter. Most females were found inside caves or at their entrance during day or night, and some females were found during the day in the leaf litter around rocky outcrops or caves. We did not observe egg laying, and juveniles were not detected. Predators of this species remain undocumented.

#### Sexual dimorphism.

Only males have prepollical nuptial excrescences. There was a significant difference between the SVL of adult males and females of *C. yucatanensis* (*t* = -9.72, df = 25, *P* < 0.05). When the effect of body length (SVL) was removed, there was a significant difference between sexes in TM (*F*_1,24_ = 11.21, *P* < 0.05), where males possess larger TM average, SL (*F*_1,24_ = 4.87, *P* < 0.05) where females are larger in average, and EN (*F*_1,24_ = 12.61, *P* < 0.05) where females possess larger EN. There was no significant difference in EL (*F*_1,24_ = 1.26, *P* > 0.05), IOD (*F*_1,24_ = 0.01, *P* > 0.05), F3 (*F*_1,24_ = 0.11, *P* > 0.05), HL (*F*_1,24_ = 3.14, *P* > 0.05), and HW (*F*_1,24_ = 0.48, *P* > 0.05) between sexes.

## Discussion

*Craugastor yucatanensis* is the only member of the genus occurring in the central and northeastern portion of the Yucatán Peninsula (Quintana Roo and Yucatán), while *C. alfredi* is known from the base of the Peninsula (Chiapas, Tabasco, and Guatemala). Increased sampling efforts in current range gaps are necessary to improve our understanding of the distribution of both species. Here we describe the previously unknown male, and document the arboreal behavior and advertisement call of *C. yucatanensis*, previously mentioned but with little detail by Calderón-Mandujano et al. (2008). We heard multiple males calling from the bushes and canopy, but the challenge of climbing trees during rain or high humidity made it difficult to capture many individuals. Similarly, Taylor (1942) mentioned the difficulty in obtaining specimens of *C. decoratus* (Taylor, 1942). Campbell (1998) noted that *C. alfredi* is usually encountered after dark, especially after rains, sitting in low vegetation in the vicinity of rocky outcroppings. Campbell et al. (1989) found the holotype of *C. polymniae* (Campbell, Lamar & Hillis, 1989) (a species with vocal sac and slits) calling from vegetation (1–3 m) at night and described its voluminous and varied vocalizations, composed of four different calls. Within the species of *Craugastor* lacking vocal slits and sac, the holotype of *C. galacticorhinus* was found calling at 18:25 h from within a hole at the base of a dirt bank, on the side of a trail; the call was described as an extremely soft single “peep” repeated about every minute (Canseco-Márquez and Smith 2004). The holotype of *C. campbelli* was found sitting on a leaf at 0.75 m above the ground on a foggy night, additional females were observed on vegetation 1 m above the ground and males 2 m above, but not calling (Smith 2005). The holotype of *C. nefrens* was found at night on a *Cecropia* leaf at 0.75 m above the ground, and additional specimens during rainy and clear nights on low vegetation or the forest floor (0.3–2.0 m), without calling (Smith 2005). Specimens of *C. cyanochthebius* were found at night on vegetation (0.25–1.0 m above the ground) in an area of outcropping limestone; the collectors heard a soft frog-like call, but the call could not be confidently associated with this species (McCranie and Smith 2006).

Here we demonstrate that *C. yucatanensis* possesses an advertisement call, despite the absence of vocal slits and sac in both sexes. Lynch (1965) mentioned that *C. yucatanensis* lacks vocal slits and sac, but curiously his sample was based only on females. Lee (1996) stated that the call of *C. yucatanensis* is unknown, and the species might be mute. The quantification of the advertisement call of *C. yucatanensis* will allow new studies of the behavior and ecology of this species, as well as comparisons with related species, and suggests that other congeners lacking vocal sacs might also vocalize. The vocal repertoire of *C. yucatanensis* that we describe in this work was recorded in situ from a population near the type locality; however, it will be essential to extend this research to different populations to identify possible intraspecific variation. *Craugastor yucatanensis* is sexually dimorphic in many characters, notably snout-vent length and tympanum diameter. Males in most of the species in the *bocourti* species series have a larger tympanum than females, with the exception of *C. spatulatus* (Smith, 1939), but we must consider that there are species where data for males are not available (e.g., *C. batrachylus* (Taylor, 1940), *C. bocourti* (Brocchi, 1877), *C. megalotympanum*, and *C. silvicola* (Lynch, 1967); Martin 1958; Campbell et al. 1989). The tympanum-to-eye ratio of all species in the series for which males are known is ≥ 50%, except for *C. galacticorhinus* (40%), *C. polymniae* (32%), and *C. spatulatus* (30%). In females, the tympanum-to-eye ratio is 43–72% (Campbell et al. 1989; Canseco-Márquez and Smith 2004). *Craugastor yucatanensis* is endemic to the Yucatán Peninsula in Mexico. Its conservation status has been evaluated as Near Threatened by the IUCN (2016), and as a species of special protection (Pr) by SEMARNAT (2010). Wilson et al. (2013) determined its Environmental Vulnerability Score as 17, placing it in the middle portion of the high vulnerability category. The description of males and advertisement call presented here should help in locating additional populations in the Yucatán Peninsula, encourage further research, and eventually generate strategies for the protection of frogs and their habitat.

## Supplementary Material

XML Treatment for Craugastor yucatanensis
